# Economic Impact of a triptan Rx-To-OTC Switch in Six EU Countries

**DOI:** 10.1371/journal.pone.0084088

**Published:** 2013-12-19

**Authors:** Aurelie Millier, Joshua Cohen, Mondher Toumi

**Affiliations:** 1 Health Economics and Outcomes Research Department, Creativ-Ceutical, Paris, France; 2 Tufts Center for the Study of Drug Development, Tufts University, Boston, Massachusetts, United States of America; 3 Unité de Formation et de Recherche d'Odontologie, Université Claude Bernard Lyon I, Lyon, France; University of New South Wales, Australia

## Abstract

**Introduction:**

Triptans have been safely and effectively used in the management of migraine for more than fifteen years, and it seems reasonable to wonder what would be the economic impact of moving a specific triptan to OTC availability. The objective of this study was then to examine the economic impact of payer policies of a triptan Rx-to-OTC switch in six EU countries (France, UK, Spain, Italy, Germany and Poland).

**Methods:**

A decision model was used to model the budgetary impact of a triptan Rx-to-OTC switch from the third-party payer (TPP) and the societal perspectives, using a one-year timeframe.

**Results:**

From the TPP perspective, it is estimated that the current overall direct spending on the management of migraine attacks across the 6 EU Member States is €582 million annually, and that the savings would reach €75 million (13% of the overall direct economic burden of migraine). From the societal perspective, €86 million annually would be added.

**Conclusions:**

Given evidence of effectiveness and safety, and given the potential savings, a triptan Rx-to-OTC switch is a reasonable public policy decision.

## Introduction

Migraine is a common, chronic neurovascular disorder characterized by severe, debilitating headaches that can last for several hours, or even days. It is often accompanied by nausea, vomiting and other neurological symptoms, such as light and noise sensitivity and visual aura. These symptoms are often disabling and affect the patient’s ability to function normally [1]. As a chronic condition, migraines recur throughout a patient's life [2].

Several migraine treatments are available: some are effective to address pain (analgesics, non-steroidal anti-inflammatory drugs (NSAIDS), and caffeine), and other aim at preventing migraines from happening. The latter include ergotamine and pethidine, but more specific migraine therapies are triptans (selective 5-hydroxytryptamine serotonin receptor agonists). Although triptans differ in their ability to prevent a recurrence of migraine, they are considered equally effective in their ability to provide relief, and are recommended as first line therapies for moderate to severe attacks [1]. However the majority of migraine sufferers still use over-the-counter (OTC) medications such as analgesics, or non-steroidal anti-inﬂammatory drugs (NSAIDs), such as aspirin, ibuprofen, or naproxen sodium [3].

It is estimated that 10% to 15% of people worldwide suffer from migraine at one point during their lives [4]. Migraine was listed by the World Health Organization (WHO) in the Global Burden of Disease Study 2000 as the 19^th^ highest cause of disability (12^th^ in women). Migraine has been acknowledged by WHO as a priority for development of effective treatments [5]. Management of the disease presents a substantial economic burden for society in terms of use of intensive health care resources (general practitioner (GP) and emergency room (ER) visits), as well as lost productivity as most sufferers are between 25 and 55 [4]. The annual cost of migraine has been estimated to be €27 billion in Europe, $US1.4 billion in the UK and $US16.6 billion in the US [6].

Medical professional societies in many countries have published clinical practice guidelines, which aim to improve the quality of care migraine patients receive by providing evidence-based recommendations to health care providers. Some guidelines target headaches of all kinds, including migraine (UK [7], Scotland [8], Croatia [9], Switzerland [10], Romania [11]). Others concern migraine alongside tension and cluster headache (Denmark [12], Italy [13]). Still others specifically target migraine management (Spain [14] and France [15]).

Primary headaches such as migraine with or without aura and episodic tension-type headache can be treated by patients themselves, without a health care provider intermediary [16]. For example, the OTC products aspirin (acetylsalicylic acid), paracetamol (acetaminophen), and non-steroidal anti-inflammatory drugs (NSAIDs) can be used to treat migraine. Nevertheless, many clinicians believe that the therapeutic class of triptans is clinically more effective for acute migraine [17]. In 2006, sumatriptan 50mg and naratriptan 2.5mg were approved as OTC drugs in the UK and Germany, respectively, following studies which demonstrated their safe and effective use in large numbers of migraine patients [18]. However, the economic impact of the switch of these two triptans in either market has not been studied to date. 

Several benefits are expected in case of an Rx-to-OTC switch for triptans. First, OTC availability will allow early, convenient access to effective therapies, in particular, the ability to self-medicate when a migraine is in its early onset stage – all medications are more effective at this stage than when the headache has progressed to one with more severe symptoms [19]. Second, easier access may result in improved outcomes and better worker productivity. Third, cost savings are expected from avoided GP and emergency room visits and lower drug prices. Nevertheless Rx-to-OTC switch has some limits, including the lack of proper monitoring that may lead to excessive use or misuse, or contraindicated use [20–23]. This may mitigate the clinical and economic benefits, unless phamarcist supervision is considered. 

Given that migraine sufferers tend to be able to self-diagnose and self-manage their condition [24], the availability of OTC triptan might be a cost-effective alternative. The main objective of this study is to assess the economic impact of a triptan Rx-to-OTC switch.

## Methods

### Study design

We estimated the impact of a potential switch of a triptan from prescription status to non-prescription status, in France, UK, Spain, Italy, Germany and Poland. 

We constructed a decision model to compare the current situation to the potential scenario in which a triptan is switched to OTC availability. The analysis was applied to several hypothetical cohorts of patients suffering from migraine. The simulations were performed from two decision-making perspectives: a third-party payer (TPP) and a societal perspective. 

The timeframe of the analysis is one year from the moment of a potential switch and represents the savings at the forecast peak uptake of the switched-to-OTC triptan. 

### Model Development

We used the checklist published by Cohen et al. [25] to develop our model, and followed their recommendations. Three distinct components of the modeling process are covered: structural issues on decision context, health states and clinical outcomes, and other considerations for model specifications.

#### 1: Structural issues on decision context

**Pathology** Migraine is a chronic condition with acute exacerbations that results in recurrent headache and patient disability. Treatments, such as the therapeutic class of triptans, are available which relieve pain and associated symptoms to allow sufferers to recover and resume normal daily activities. While triptans are only taken during the migraine attacks (therefore not pre-emptively or prophylactically), their timely uptake is crucial, as the literature suggests that the earlier treatment is taken the better is the therapeutic effect [26]. Results from three retrospective analyses of the effectiveness of early treatment with sumatriptan shows that early treatment (while pain is generally still mild) significantly improves the pain free response [19]. These studies point to an additional benefit: fewer patients require treatment re-dosing, which translates into less medication use and consequently lower pharmaceutical costs.
**Triptan class of drugs** The long term safety of triptans has been validated [27]. Even in instances in which triptans are contraindicated in patients presenting with cardiac or cerebrovascular diseases, triptans have been shown to be safer than other treatment alternatives, such as serotonin-agonist ergot preparations, morphine or pethidine[18]. Moreover, other options may pose a risk to certain patients, as aspirin and non-steroidal anti-inflammatory drugs may cause gastro-intestinal hemorrhage [18].
**Populations** Among the population suffering from migraine, six subgroups were selected: Pop 1 (diagnosed, treated with Rx triptan to be switched), Pop 2 (diagnosed, treated with other Rx triptans), Pop 3 (diagnosed, treated with OTC), Pop 4 (diagnosed untreated), Pop 5 (undiagnosed, treated with OTC), Pop 6 (undiagnosed, untreated). Population of patients being prescribed Rx drugs other than triptans were not included.

#### 2: Health states and clinical outcomes

**Epidemiology** Country-specific data are available on national statistics websites, which may be complemented by trade- and peer-reviewed literature searches.
**Health outcomes** Given that the main objective of this analysis is to estimate the economic impact of a triptan Rx-to-OTC switch, and not the health benefit, the model did not include health outcomes. On the other hand, serious adverse events were considered, in particular, cardiovascular events that may occur when undiagnosed patients switch to OTC triptan. Only economic impact of serious adverse events was included in the model.
**Resource utilisation** The model includes direct and indirect costs. Direct costs comprise drug acquisition costs, cost of GP and ER visits, as well as costs due to serious adverse events. Indirect costs refer to productivity losses owing to time off from work.
  From the TPP perspective, the model includes direct costs savings (less money spent on Rx drug acquisition, fewer GP and ER visits due to easier access to an effective therapy). The societal perspective also includes indirect costs savings (less absenteeism and improved employee productivity).

#### 3: Other considerations for model specifications

**Patient behaviour** The model assumes that the patient will not require a doctor visit with the OTC drug.
**OTC switch rate** There is no published evidence on switch rates for triptans in migraine. Therefore, assumptions were made based on estimates from previous examples of Rx-to-OTC switches in Europe. Here, it was necessary to distinguish between patients currently taking Rx triptan to be switched, those on any other triptan, those taking OTC drugs at the time of migraine attacks, and those not using any pharmaceutical agents. The base-case model assumes that following the switch, the switched triptan retains a ‘dual reimbursement status’ (available without a prescription and not reimbursed, or reimbursed if prescribed).
**Misuse** Medication-overuse headache (MOH) is a serious and disabling disorder. We considered the implications of MOH in the model in terms of additional GP visits and additional productivity losses.

### Input data

Input data include epidemiological and resource utilization data.

#### 1: Epidemiological Data

**Populations** This section presents the methodology used to estimate the numbers of patients of each sub-population. First we estimated the total country population aged over 18, and applied a country-specific migraine prevalence rate to calculate disease incidence. Then we used a diagnosis rate, to distinguish between the diagnosed and the undiagnosed patient populations. We created a treatment algorithm based on publically available prescription patterns as well as sales data. For diagnosed patients, we identified treated and untreated migraine patients. Subsequently, for the treated population, we estimated the population sizes depending on their treatment strategies (Rx, both Rx and OTC, or untreated). Finally, by combining these estimates with sales data of triptan to be switched and other Rx triptans, we ended up with an approximate size of patients in Pop 1, Pop 2, Pop 3, and Pop 4. For the undiagnosed patients, we assumed that the percentage of treated patients (OTC, self-treated) corresponds to the percentage of those diagnosed and treated. This allowed an estimation of the size of Pop 5 and Pop 6.
**Switch rates** Switch rates were mainly based on estimates from past examples of Rx-to-OTC switches in Europe. These were obtained from internal pharmaceutical sales data sources. In the base case scenario we assumed the peak uptake of OTC triptan to be switched would be 20% among both patients currently taking the Rx triptan and those on any other triptan, and 3% among patients either diagnosed with triptan but currently taking OTC drugs at the time of migraine attacks, or not using any pharmaceutical treatments. No distinction was found in the literature to support a country-specific switch rate.
Table 1 presents the epidemiological inputs included in the model.

**Table 1:** Epidemiological inputs included in the model.

	**France**	**UK**	**Italy**	**Germany**	**Poland**	**Spain**	**Source**					
							**France**	**UK**	**Italy**	**Germany**	**Poland**	**Spain**
**Population**												
Population size	62,616,488	61,792,000	60,221,211	81,879,976	38,149,886	45,957,671	[36]	[38]	[36]	[36]	[36]	[36]
Population over 18 years old	78%	77%	83%	84%	81%	82%	[37]	[38]	[39]	[40]	[41]	[42]
Migraine prevalence	21.3%	15%	11.6%	10.0%	10.0%	12.6%	[43]	[35]	[44]	[45]	[46]	[47]
Adults with migraine population	10,403,103	7,136,976	5,798,098	6,877,918	3,090,141	4,748,347	-	-	-	-	-	-
**Diagnosis rates and prescription patterns**												
Diagnosis rate	75%	65%	50%	50%	50%	50%	[48]	[49]	[50]	[50]	[50]	[50]
Diagnosed patients	7,802,327	4,639,034	2,899,049	3,438,959	1,545,071	2,374,174	-	-	-	-	-	-
% treated	90%	89%	90%	90%	90%	90%	[51]	[52]	[51]	[51]	[51]	[51]
Treated patients	7,022,094	4,128,740	2,609,144	3,095,063	1,390,564	2,136,757	-	-	-	-	-	-
% adult treated with Rx (w or w/o OTC drugs)	50%	41%	50%	50%	50%	50%	[51]	[49]	[51]	[51]	[51]	[51]
Adults treated with Rx (w or w/o OTC drugs)	3,511,047	1,692,783	1,304,572	1,547,532	695,282	1,068,379	-	-	-	-	-	-
% adult treated with triptan	25%	22%	19%	31%	5%	18%	[53]	[53]	[53]	[53]	[53]	[53]
% adult treated with Rx triptan to be switched	35%	17%	9%	23%	3%	29%	[53]	[53]	[53]	[53]	[53]	[53]
Pop 1 (diagnosed, treated with Rx triptan to be switched)	309,354	62,793	21,634	109,646	966	55,942	-	-	-	-	-	-
Pop 2 (diagnosed, treated with other Rx triptans)	563,206	313,181	232,084	375,642	30,627	139,250	-	-	-	-	-	-
Pop 3 (diagnosed, treated with OTC)	3,511,047	2,435,957	1,304,572	1,547,532	695,282	1,068,379	-	-	-	-	-	-
sPop 4 (diagnosed, untreated)	780,233	510,294	289,905	343,896	154,507	237,417	-	-	-	-	-	-
Undiagnosed patients	2,600,776	2,497,942	2,899,049	3,438,959	1,545,071	2,374,173	-	-	-	-	-	-
% treated with OTC (only)	90%	89%	90%	90%	90%	90%	[51]	[49]	[51]	[51]	[51]	[51]
Pop 5 (undiagnosed, treated with OTC)	2,340,698	2,223,168	2,609,144	3,095,063	1,390,563	2,136,756	-	-	-	-	-	-
Pop 6 (undiagnosed, not treated)	260,078	274,774	289,905	343,896	154,507	237,417	-	-	-	-	-	-
**Switch rates**												
Pop 1 (diagnosed, treated with Rx triptan to be switched)	20%											
Pop 2 (diagnosed, treated with other Rx triptans)	20%						Base case assumption					
Pop 3 (diagnosed, treated with OTC)	3%											
Pop 4 (diagnosed, untreated)	3%											
Pop 5 (undiagnosed, treated with OTC)	0%											
Pop 6 (undiagnosed, not treated)	0%											
**Adverse events**												
% of decrease in the risk of adverse events (thanks to label indications, pharmacist training) for Pop 3, Pop 5 and Pop 6	95%						Base case assumption					

#### 2: Resource Utilization

Costs have been calculated depending on the population involved and the perspective selected. This section describes our calculation methods, the assumptions used, and Table 2 presents the main inputs used.

**Table 2:** Resource utilization inputs included in the model.

	**France**	**UK**	**Italy**	**Germany**	**Poland**	**Spain**		**Source**					
								**France**	**UK**	**Italy**	**Germany**	**Poland**	**Spain**
**Disease related assumption**													
Incidence of migraine attacks per patient per year	24							[54]					
**Drug acquisition costs and reimbursement levels**													
Triptan to be switched unit cost per attack	€7.36	£6.92	€9.65	€6.46	PLN 16.6	€11.22		Calculation					
Other Rx triptans unit cost per attack	€6.12	£6.09	€8.05	€5.59	PLN 19.87	€9.25		Calculation					
Reimbusement rate triptan to be switched	65%	100%	100%	100%	0%	100%		IMS data					
Reimbusement rate other Rx triptans	65%	100%	100%	100%	0%	100%		IMS data					
**GP visits**													
Number of GP visits specifically for migraine per patient per year	2.8							[30]					
% of unscheduled visits (i.e. visits exclusively to obtain prescription)	30%							[55]					
Cost of a GP visit	€22	£39	€21	€31	PLN 31	€23		[56]	[57]	[58]	[59]	[60]	[61]
**Emergency room visit costs**													
Probability of ER visit per attack when treated with usual care (%)	0.4%							[31]					
Reduction in ER visits with triptans	65%							[31]					
Cost of migraine ER visit	€25	£51	€62	€53	PLN 147	€128		[62]	[57]	[55]	[63]	[64]	[65]
**Serious adverse events**													
% of triptan contraindicated patient	8%							[32]					
Incidence of serious adverse event	0.1%							[33]					
% of decrease in the risk of serious AEs (thanks to label indications, pharmacist trainings)	95%							Expert assumption					
Average cost of a serious cardiovascular event	€1,694	£1,916	€3,416	€3,610	PLN 2,927	€4,320		[66]	[67]	[68]	[69]	[70]	[71]
**Productivity costs**													
Number of days with migraine attack per patient per year (Pop 1 and Pop 2)	24							[54]					
Number of days with migraine attack per patient per year (Pop 3,Pop 4, Pop 5 and Pop 6)	12							[35]					
% of attacks during work hours	35%							[72]					
Probability of not having triptan at time of attack	25%							[72]					
Absenteeism													
% of migraine attacks work days resulting in absenteeism (Pop 1 and Pop 2)	24%							[73]					
% of migraine attacks work days resulting in absenteeism (Pop 3, Pop 4, Pop 5 and Pop 6)	8%							[35]					
Presenteeism													
% of migraine attacks with symptoms affecting patient productivity (Pop 1 and Pop 2)	73%							[35]					
% of migraine attacks with symptoms affecting patient productivity (Pop 3, Pop 4, Pop 5 and Pop 6)	24%							[35]					
Productivity level of migraine patient while suffering from symptoms	56%							[74]					
% increase in productivity during migraine attacks related to triptan use	13%							[74]					
**Avoided time off work**													
% of employed migraine sufferers	70%							[30]					
Average time missed due to a doctor visit (work days)	0.5							[34]					
% of visits made during work hours	25%							[34,75]					
Average cost of one lost work day to the economy	€127	£120	€107	€164	PLN 180		€94	[76]	[77]	[78]	[79]	[80]	[78]
**Medication-overuse headache (MOH)**													
% of triptan users likely to experience MOH	14%							[81]					
% of reduction in the risk of MOH thanks to smaller OTC packages and pharmacist training	95%							Expert assumption					
Additional number of GP visits due to MOH per patient per year	8.4							[82]					
Additional number of days with migraine attack per MOH patient per year	108							Calculated from [81] and [83]					
Absenteism (% of lost work days resulting in absenteeism due to MOH)	24%							[84]					
Presenteism (% of migraine attacks in patients with MOH with symptoms affecting patient productivity	55%							[84]

**Drug acquisition costs** Average cost per attack was calculated as cost per dose of triptan multiplied by mean doses per attack, weighted according to market share [28]. Cost per dose has been identified as being the drug acquisition cost divided by the number of doses in the pack. Mean dose by attack was set at 1.61, as stated by Guidotti et al. [29]. Reimbursement rate for all triptans by payers (without co-payment) was 100% before the switch Germany, Spain and Italy, 65% in France and 0% in Poland (0%).
**GP visits** Number of migraine-specific GP visits per year was found to be 2.8 from a 2001 multinational survey (US and Europe) using a self-reported Migraine Background Questionnaire [30]. Of these visits, 30% were assumed to be unscheduled visits to obtain a prescription. We used country-specific unit costs of GP visits.
**Emergency room visit costs** The probability of ER visit per attack when treated with usual care was found to be 0.4%, and the reduction in ER visits with triptan was found to be 65% [31]. We used country-specific unit costs of ER visits.
**Serious adverse events** Percentage of patients presenting at least one contraindication to triptan was found to be 8% in a retrospective claims analysis study conducted in France [32]. Serious adverse events represented an overall incidence of 0.1% [33]. The percentage of decrease in the risk of serious AE thanks to label indications, and/or pharmacists training was assumed to be 95%. We used country-specific costs of serious AE.
**Time off from work** We derived the estimated percentage (70%) of employed migraine patients from a survey conducted in 23 US and 78 non-US sites [30]. The average time missed due to a GP visit was derived from the Association of Great Britain [34], and was estimated to be 0.5 work days. We also used their estimation of percentage of visits made during working hours (25%). The average cost of one working day was country-specific. Cost savings due to avoided absenteeism was then computed as the number of days missed from work due to GP visits per year multiplied by the average country-specific cost of one working day.
**Productivity losses** Cost savings due to avoided productivity losses were defined as number of missed work days avoided and of avoided missed work days exclusively lost due to presenteeism, multiplied by the average cost of one lost work day, weighted by the increase in work effectiveness during migraine attacks related to triptan use.
  The percentage of migraine attacks on work days that resulted in absenteeism was estimated to be 24% (a survey among English patients reported the average number of migraine attacks to be 24 and the average number of days lost to be 5.7 [35]. The same source indicated that the percentage of migraine attacks on work days with symptoms affecting patients’ productivity was estimated to be 73%.

### Sensitivity analyses

We depicted several scenarios to illustrate how the potential cost savings would vary depending on different switch rates. 

#### Scenarios A and B

Given the uncertainties and the lack of relevant publically available evidence about past switches, switch rates were varied to analyze the potential impact on results. In scenario A switch rates for Pop 1 and Pop 2 were decreased to 0% instead of 20%. In scenario B switch rates for Pop 5 and Pop 6 were increased to 3% instead of 0%. This is summarized in Table 3.

**Table 3:** Scenarios around the switch rates.

**Population**	**Base case scenario**	**Scenario A (switch rates)**	**Scenario B (switch rates)**
Pop 1 (diagnosed, treated with Rx triptan to be switched)	20%	0%	20%
Pop 2 (diagnosed, treated with other Rx triptans)	20%	0%	20%
Pop 3 (diagnosed, treated with OTC)	3%	3%	3%
Pop 4 (diagnosed untreated)	3%	3%	3%
Pop 5 (undiagnosed treated with OTC)	0%	0%	3%
Pop 6 (undiagnosed not treated)	0%	0%	3%

#### Scenario C

Clear labeling and pharmacists training were assumed to decrease cardiovascular event risk from 95%. This rate was varied to 90%, to illustrate the importance of incorporating measures/policies that could minimize any potential risk of misuse.

#### Scenario D

The impact of non-coverage policies for OTC products was evaluated: following the switch, the Rx status of the switched triptan was removed.

### Software

The model was developed/designed in Microsoft Office Excel 2007.

## Results

Savings calculated by the model are presented in Table 4. From the TPP perspective, the model estimates that the current overall direct spending on the management of migraine attacks across the 6 EU Member States is €582 million annually. That includes the cost of reimbursed triptans, GP visits and ER visits. The estimated annual direct savings to public healthcare budgets associated with switching are estimated to be €75 million accounting for 12.9% of the overall direct economic burden of migraine (€21.2 million in France, £13.9 million in UK, €11.0 million in Italy, €16.1 million in Germany, PLN 0.3 million in Poland and €10.2 million in Spain). From the societal perspective, current overall spending on migraine management is €3,489 million annually, and estimated savings are estimated at €86 million annually, accounting for 2.5% of the budget (€25.4 million in France, £15.7 million in UK, €12.0 million in Italy, €19.0 million in Germany, PLN 0.7 million in Poland and €10.9 million in Spain).

**Table 4:** Base case analysis results.

		**Direct costs/savings**					**Indirect costs/savings**				**TOTAL costs**	**TOTAL costs**
		**Drug acquisition**	**GP visit**	**ER visits**	**CV events**	**MOH GP visit**	**Time off work**	Productivity loss (Pop1 & Pop2)*	Productivity loss (Pop3 & Pop4)**	**Additional MOH productivity costs**	**TPP perspective**	**Societal perspective**
France	Costs before the switch	89,283,831	53,749,696	10,426,799			27,149,704	361,321,298	475,922,844		153,460,326	1,017,854,172
(€2010)	Costs after the switch	71,427,065	50,524,714	10,257,312	872	30,279	25,520,722	359,510,261	474,983,011	233,411	132,240,243	992,487,648
	Total savings	17,856,766	3,224,982	169,486	-872	-30,279	1,628,982	1,811,037	939,832	-233,411	21,220,083	25,366,524
	Costs before the switch	56,233,629	41,056,361	15,168,153			11,022,317	146,690,285	354,379,442		112,458,143	624,550,187
UK (£2010)	Costs after the switch	44,986,903	38,592,979	14,967,686	677	35,106	10,360,978	145,955,035	353,771,477	143,834	98,583,351	608,814,675
	Total savings	11,246,726	2,463,382	200,467	-677	-35,106	661,339	735,250	607,965	-143,834	13,874,792	15,735,512
Italy	Costs before the switch	49,865,795	14,705,495	14,882,863			6,651,217	88,517,593	261,829,545		79,454,153	436,452,509
(€2010)	Costs after the switch	39,892,636	13,823,166	14,741,253	654	10,586	6,252,144	88,073,920	261,535,332	73,069	68,468,294	424,402,759
	Total savings	9,973,159	882,330	141,610	-654	-10,586	399,073	443,673	294,213	-73,069	10,985,859	12,049,750
Germany	Costs before the switch	67,371,768	41,905,589	16,029,650			19,451,314	258,867,418	474,886,315		125,307,007	878,512,054
(€2010)	Costs after the switch	53,897,414	39,391,254	15,855,573	819	18,709	18,284,235	257,569,907	474,352,694	132,527	109,163,769	859,503,132
	Total savings	13,474,354	2,514,335	174,077	-819	-18,709	1,167,079	1,297,512	533,621	-132,527	16,143,238	19,008,923
Poland	Costs before the switch		2,742,272	17,343,966			1,393,251	18,542,057	234,747,009		20,086,238	274,768,556
(PLN 2010)	Costs after the switch		2,577,736	17,212,552	298	8,449	1,309,656	18,449,119	234,483,229	65,511	19,799,035	274,106,550
	Total savings		164,536	131,414	-298	-8,449	83,595	92,938	263,781	-65,511	287,203	662,006
Spain	Costs before the switch	45,988,181	12,346,284	24,924,237			4,491,924	59,780,684	188,233,144		83,258,702	335,764,454
(€2010)	Costs after the switch	36,790,545	11,605,507	24,690,627	677	9,461	4,222,409	59,481,047	188,021,630	52,530	73,096,817	324,874,433
	Total savings	9,197,636	740,777	233,609	-677	-9,461	269,515	299,637	211,514	-52,530	10,161,885	10,890,021
Total	Costs before the switch	320,260,935	172,846,336	88,799,849			71,366,381	949,778,064	1,885,512,429		581,907,120	3,488,563,994
(€2010) ***	Costs after the switch	256,208,748	162,475,556	87,807,250	3,911	113,407	67,084,399	945,017,528	1,882,735,949	680,927	506,608,872	3,402,127,675
	Total savings	64,052,187	10,370,781	992,597	-3,911	-113,407	4,281,982	4,760,537	2,776,479	-680,927	75,298,247	86,436,318

^*^ Productivity loss due to GP visits to get medication and due to migraine attacks for Pop 1 & Pop 2

^**^ Productivity loss due to migraine attacks for Pop 3 & Pop 4

^***^ All costs have been converted into €2010, using with the following rates: €1 = PLN 4.07 PLN (06/2010), €1= £ 0.83 (06/2010)

CV=cardiovascular, GP=General Practitioner, MOH=medication over use

The majority of savings (85% for TPP perspective and 74% from societal perspective) comes from drug acquisition costs that are shifted from payers to patients. GP visits avoided contribute for 14% of direct savings and ER visits avoided for 1%. Additional cardiovascular events and additional GP visits due to MOH had a negligible impact on budget. 

When considering the societal perspective, productivity loss due to migraine management (GP visits to get medication and due to migraine attacks for Pop 1 & Pop 2, and due to migraine attacks for Pop 3 & Pop 4) or account for 81% of current spending.

The additional cost implications resulting from a (potential) slight increase in the risk of AE due to a lack of doctor’s supervision are negligible: €0.11 million in direct costs plus an estimated €0.68 million of productivity losses, mainly from additional physician visits from patients that will develop MOH.

The employer benefits account for a total of 14% (or €11 million) and are similar in size to the savings that result from avoided GP visits. There are 3 major sources of the employer benefits:

• Time taken off work for patients to obtain a prescription for Rx triptan to be switched- sick leave
• Productivity gains due to convenient access to switched triptan for patients who previously had to obtain a prescription for switched triptan
• Productivity gains due to increased access to switched triptan in untreated patients or those currently treated with an OTC product

### Sensitivity analysis

The model has shown to be particularly sensitive to switch rates across different patient groups. In scenario A it was assumed that diagnosed patients treated with Rx triptan (Pop 1 & Pop 2) would not switch to OTC triptan to be switched and thus switch rates were decreased to 0% instead of 20%. An unfavorable impact on the results is found for all countries. For example, in France, the savings fell from 13.83% and 2.49% to 0.05% and 0.08% for TPP and societal perspectives respectively.

In scenario B, where it was assumed that undiagnosed patients would switch to OTC triptan to be switched: switch rates for undiagnosed populations (Pop 5 & Pop 6) were increased to 3% instead of 0%, a favorable impact on the results is seen for all countries. As an example, in the UK, the savings increased from 12.34% to 12.43% for TPP perspective and from 2.52% to 2.61% for societal perspective.

The results of the scenario C suggest the model is insensitive to the costs of cardiovascular events due to its rare frequency. Considering 90% decrease instead of 95% decrease in risk of cardiovascular event has a negligible impact.

Finally, the scenario D shows that the introduction of the disreimbursement policies resulted in additional drug savings to the healthcare budget holder. Overall, savings increased from €75 million to around €86 million for TPP perspective and from €86 million to around €100 million from societal perspective.

## Discussion

A triptan Rx-to-OTC switch represents a way for payers to manage spending. This is particularly relevant at a time of cash-strapped healthcare budgets across Europe. Seventy-five percent of the cost savings (€64 million) come from shifting drug costs from payers to patients. Such savings are easily and quickly realized. In contrast, other cost savings such as avoided GP or ER visits, although important, are not necessarily easy to realize in some EU countries as primary care doctors are paid a flat annual fee regardless of the number of patient visits.

Enhanced availability of OTC medicines can provide patients easier access to effective and safe drugs, implying a reduction in the number of days with significantly impaired work productivity across the 6 EU Member States. Our model estimates that 830,000 patients of all migraine sufferers will be purchasing the OTC triptan when it reaches the forecast peak sales. Almost half of the usage of the OTC triptan (46%) is estimated to be among patients that are currently treated with partially effective OTC analgesics or those who are not treated at all. It is possible to further reduce the disease burden, if manufacturers invest in a disease awareness campaign that will promote awareness of the condition to currently undiagnosed patients (40% of all migraine sufferers).

Given that the majority of cost savings come from reduced spending by payers on triptans, the highest level of savings would be generated in countries where triptans are currently reimbursed at 100% without patient co-payments. In countries where triptans are not reimbursed, such as Poland, the economic benefits would be non-negligible but far lower. 

The model estimates that the current overall direct spending on the management of migraine attacks across the 6 EU Member States is €582 million. That includes the cost of reimbursed triptans, GP visits and ER visits. The estimated direct savings associated with switching one triptan are estimated to be €75 million accounting for 13% of the overall direct economic burden of migraine.

Should the price of OTC triptan be attractive, we estimate that 20% of patients currently taking the Rx triptan product will switch to OTC triptan, and the societal economic benefits will be around €86 million in the 6 EU Member States. In case patients perceive the OTC price as being too high, they may prefer to visit a primary care doctor to obtain a prescription and pay only a (nominal) co-payment or a dispensing fee for the reimbursed product.

Several limitations are to be noted. First, our model did not include implications of a possible misdiagnosis of serious disease and wastage of resources in patients for whom OTC treatment is inappropriate. This model also assumed the switch rate would be the same for all countries, as no relevant literature providing evidence of country-specific behaviour was found. Finally, it is not excluded that there might be a perverse incentive; currently patients are used to getting ‘free triptan’ from ‘free GP visit’, but after the Rx-to-OTC switch, they will have to pay to get the OTC medicine. This may incite patients to be prescribed more expensive ‘free’ medicine (like higher doses of triptan, other Rx triptans, or even morphine). All these points were discussed with experts and impact was assumed to be negligible.

## Conclusions

The results of the model suggest that the switch of a triptan to the OTC status is likely to result in substantial economic benefits of which the majority could be quickly realized by payers across the 6 investigated EU countries. Around 74% of the economic benefits come from lower spending on the Rx triptan when considering societal perspective. These savings are likely to be bigger is all 27 EU member states are considered. Given that the majority of the savings come from the avoided drug acquisition cost, they would be mainly generated in countries where the triptan is currently reimbursed.
